# Supplementary material for: A KDM6A–KLF10 reinforcing feedback mechanism aggravates diabetic podocyte dysfunction
Source: EMBO Mol Med. 2019 Apr 4;11(5):e9828. doi: 10.15252/emmm.201809828 (PMC6505577; doi:10.15252/emmm.201809828)
Supplement: Supplementary file 1 — Appendix [file EMMM-11-e9828-s001.pdf]

APPENDIX

A KDM6A–KLF10 REINFORCING FEEDBACK MECHANISM AGGRAVATES DIABETIC PODOCYTE DYSFUNCTION

TABLE of CONTENTS

Figure S1–Analysis of the DNA methylation status at the nephrin gene promoter by methylation-specific PCR ……………………..…………………………………………………….4

Figure S2–Association of changes in the expression levels of KDM6A and neprhin in kidney with the development of proteinuria in STZ-treated diabetic mice……..………………….......6

Figure S3–In vivo analysis of diabetic mice treated with a KDM6A inhibitor GSK-J4……..........7

Figure S4–Genotyping and expression of KDM6A in KDM6A-KO mice ……………………......8

Table S1–Exact P values for Figure 1……………….……...…………………..........................9

Table S2–Exact P values for Figure 2…………………....…………………..........................10

Table S3–Exact P values for Figure 3…………………..……………………..........................12

Table S4–Exact P values for Figure 4…………………..……………………..........................14

Table S5–Exact P values for Figure 5…………………..……………………..........................16

Table S6–Exact P values for Figure 6…………………..……………………..........................19

Table S7–Exact P values for Figure 7…………………..……………………..........................21

Table S8–Exact P values for Figure EV1………………..……………………........................22

Table S9–Exact P values for Figure EV3………………..……………………........................23

Table S10–Exact P values for Figure EV4..……………...…………………….......................24

Table S11–Exact P values for Figure EV5………………...…………………….......................26

Table S12–Baseline characteristics of control subjects and patients with diabetic nephropathy…..28

A

MethPrimer result

(mouse nephrin gene promoter: 2000 bp)

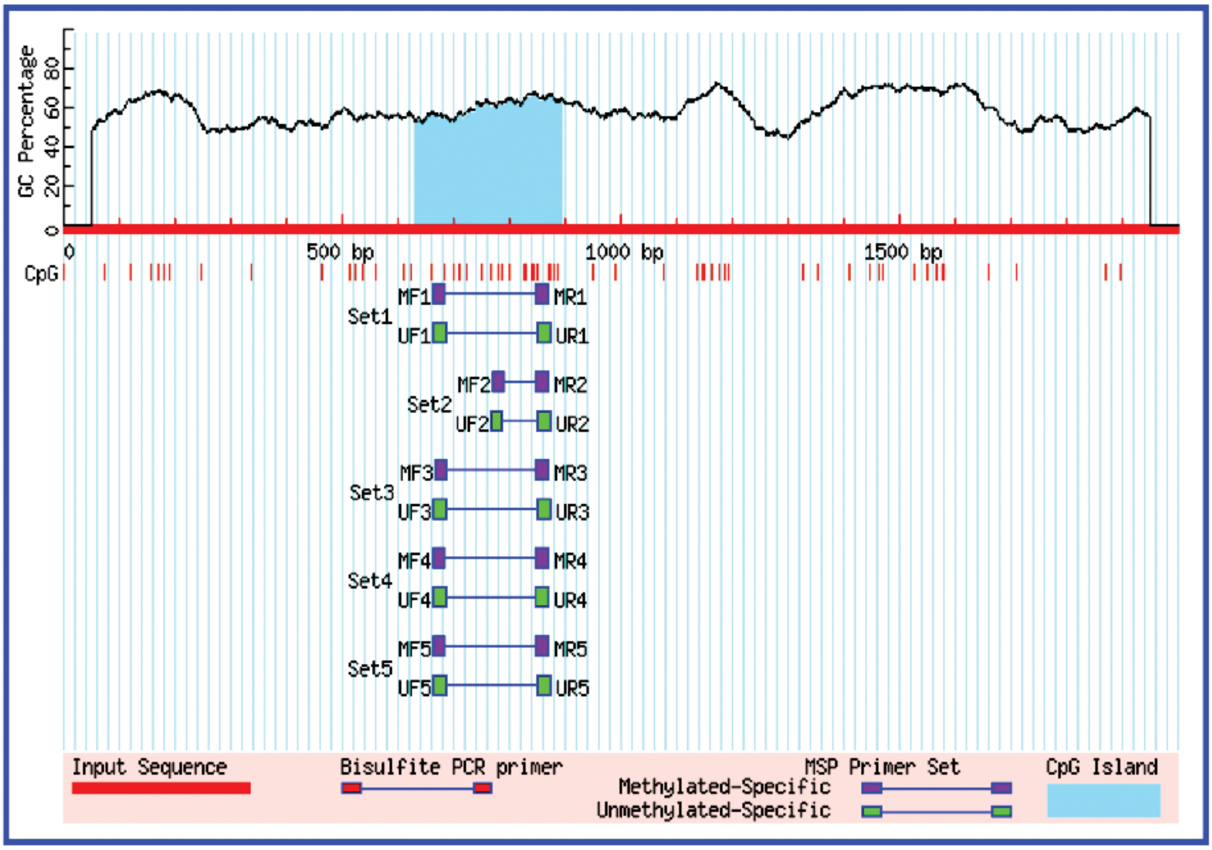

B

Primer picking results for methylation specific PCR (MSP)

	Primer	Start	Size	Tm	GC%	'C's	Sequence
1	Left M primer	665	23	58.01	52.17	4	GGGTTGGAAGATTTTATGTTTTC
	Right M primer	873	24	58.57	58.33	4	GAACAACTACCCTAAACATCCGTA
	Product size: 209, Tm: 67.8						
	Left U primer	666	24	58.53	50.00	4	GGGTTGGAAGATTTTATGTTTTTGA
	Right U primer	874	25	58.50	60.00	4	CAAACAACTACCCTAAACATCCATA
	Product size: 209, Tm: 66.4						
2	Left M primer	770	22	58.57	63.64	6	GGAGGAATTTTATTCGTTTCGT
	Right M primer	873	24	58.57	58.33	4	GAACAACTACCCTAAACATCCGTA
	Product size: 104, Tm: 63.6						
	Left U primer	769	23	57.62	65.22	6	GGGAGGAATTTTATTTGTTTTGT
	Right U primer	874	25	58.50	60.00	4	CAAACAACTACCCTAAACATCCATA
	Product size: 106, Tm: 63.1						
3	Left M primer	667	23	57.40	47.83	4	GTTGGAAGATTTTATGTTTTCGA
	Right M primer	873	24	58.57	58.33	4	GAACAACTACCCTAAACATCCGTA
	Product size: 207, Tm: 67.9						
	Left U primer	666	24	58.53	50.00	4	GGTTGGAAGATTTTATGTTTTTGA
	Right U primer	874	25	58.50	60.00	4	CAAACAACTACCCTAAACATCCATA
	Product size: 209, Tm: 66.4						
4	Left M primer	665	23	58.01	52.17	4	GGGTTGGAAGATTTTATGTTTTC
	Right M primer	873	24	58.57	58.33	4	GAACAACTACCCTAAACATCCGTA
	Product size: 209, Tm: 67.8						
	Left U primer	666	24	58.53	50.00	4	GGTTGGAAGATTTTATGTTTTTGA
	Right U primer	873	24	56.06	58.33	4	AAACAACTACCCTAAACATCCATA
	Product size: 208, Tm: 66.5						
5	Left M primer	665	23	58.95	52.17	4	GGTTGGAAGATTTTATGTTTTCG
	Right M primer	873	24	58.57	58.33	4	GAACAACTACCCTAAACATCCGTA
	Product size: 208, Tm: 67.9						
	Left U primer	666	24	58.53	50.00	4	GGTTGGAAGATTTTATGTTTTTGA
	Right U primer	874	25	58.50	60.00	4	CAAACAACTACCCTAAACATCCATA
	Product size: 209, Tm: 66.4						

C

```
          U>>>>> >>>>>>>>>> >>>>>>>>>
           M>>>> >>>>>>>>>> >>>>>>>>>
661 CTCCGGGCTG GAAGATTTTA TGTCCCCGAA ATCTGGCTCA TCCGCAGTCT CCGGTGTTCT
721 CTACCGCTCA AGGGTTGCAA CCCTAGGACT GCGAGGGACC CACTAGGCGG GAGGAATCCT
781 ATTCGCCCCG CCTCCTAGAG TCCCGGCCTC TTCCCATTGA GAAACTCCCG CGCCTTGTGT
841 GCGGACGGCT ACGGATGCTC AGGGTAGCTG TCCGCGCAGC AGGCGGGCGC CCCTGCATTA
             < <<<<<<<<< <<<<<<<<<< <<<<U
             < <<<<<<<<< <<<<<<<<<< <<<M
```

D

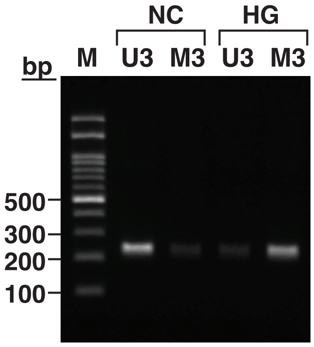

Appendix Figure S1 – Analysis of the DNA methylation status at the nephrin gene promoter by methylation-specific PCR.

A CpG island prediction results. A 2000-bp nephrin promoter region (NC_000073.6; Mouse strain C57BL/6J chromosome 7, GRCm38.p4) was used in CpG island prediction by MethPrimer (http://www.urogene.org/methprimer). A CpG island was found within the promoter region from nt 633 to 895. The criteria used for defining CpG island include the DNA length >100 bp, GC percent > 50.0, and observed/expected CpG ratio > 0.6.

B Primer picking results for methylation-specific PCR. Primer sets of PCR-based methylation analysis were designed following bisulfite conversion of DNA template.

C DNA sequence of the selected nephrin gene promoter region and localization of specific primer sets (U3 and M3) used in the methylation-specific PCR experiments. The unmethylated-specific primers (U3) include 5’-GGTTGGAAGATTTTATGTTTTTGA and 5’-CAAACAACTACCCTAAACATCCATA, whereas the methylated-specific primers (M3) include 5’-GTTGGAAGATTTTATGTTTTCGA and 5’-GAACAACTACCCTAAACATCCGTA.

D Representative methylation-specific PCR analysis of the nephrin gene promoter using U3- and M3-specific primer sets. After bisulfite conversion of genomic DNAs from immortalized podocytes cultured in normal (5 mM) or high glucose (30 mM), PCR amplification was performed using U3 or M3 primer sets. Presented experiments were performed at least three times independently.

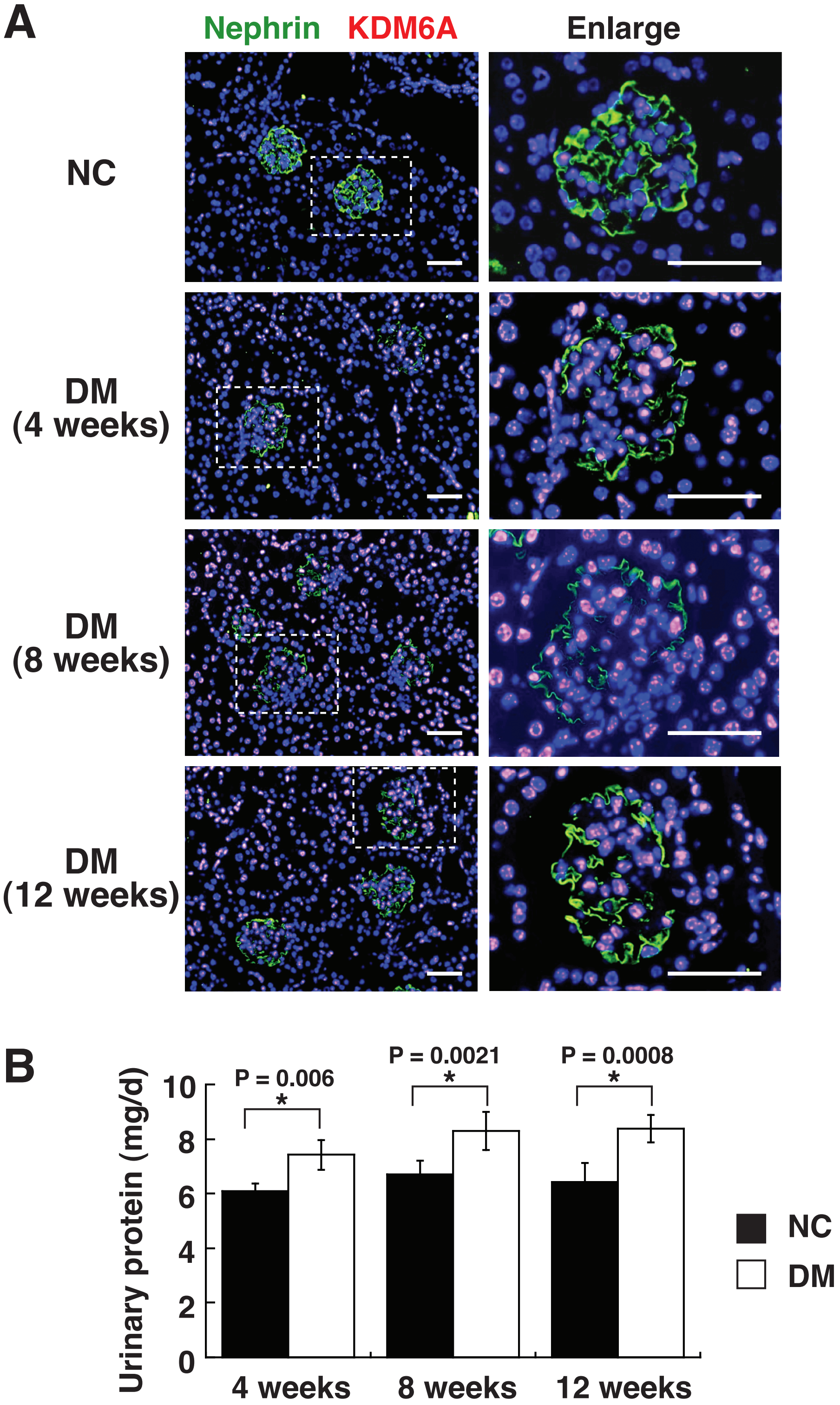
A
Nephrin KDM6A
Enlarge
NC
DM
(4 weeks)
DM
(8 weeks)
DM
(12 weeks)
B
Urinary protein (mg/d)
P = 0.006
P = 0.0021
P = 0.0008
*
*
*
0
2
4
6
8
10
4 weeks
8 weeks
12 weeks
NC
DM

Appendix Figure S2 – Association of changes in the expression levels of KDM6A and nephrin in kidney with the development of proteinuria in STZ-treated diabetic mice.

A Double immunofluorescence staining of mouse kidney sections with nephrin (green) and KDM6A (red). Kidney samples from normal mice and the 4-, 8-, and 12-week diabetic mice were included in the experiment. Right panels are enlarged views of the areas enclosed with dashed boxes shown in left panels. Scale bars, 50 μm. Compared to control kidney sections, both decreased nephrin expression and increased KDM6A expression were substantially observed in kidney sections of the 4-, 8-, and 12-week diabetic mice. Experiments were repeated at least twice.

B Levels of urinary protein excretion in normal and diabetic mice. Urinary total protein excretion was measured at 4, 8 and 12 weeks after diabetic induction. *$P < 0.05$ versus normal controls (Wilcoxon two-sample test; $n = 8$).

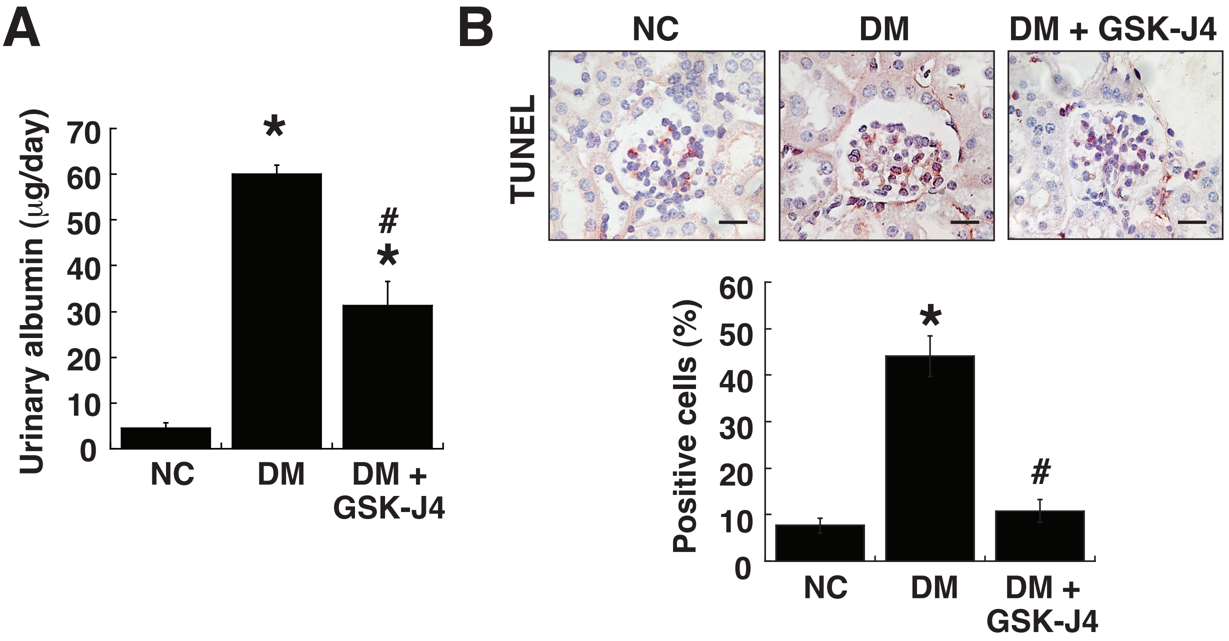

Appendix Figure S3 – *In vivo* analysis of diabetic mice treated with a KDM6A inhibitor GSK-J4.

A Urinary albumin excretion in normal, diabetic and GSK-J4-treated diabetic mice. Urinary albumin levels were measured with a turbidimetric immunoassay (Autokit Micro Albumin, Wako, Osaka, Japan) at 12 weeks after diabetic induction. *$P < 0.05$ versus normal controls, $^{\#}P < 0.05$ versus untreated diabetic mice (Parametric ANOVA and a Bonferroni *post hoc* test; $n = 8$).

B Detection of glomerular cell apoptosis in normal, diabetic and GSK-J4-treated diabetic mice. TUNEL assay for apoptosis was performed using an assay kit according to the manufacturer's instruction (#TAAP01D, BioTnA Biotech., Kaohsiung Taiwan). Scale bars, 20 μm. *$P < 0.05$ versus normal controls, $^{\#}P < 0.05$ versus untreated diabetic mice (Parametric ANOVA and a Bonferroni *post hoc* test; $n = 3$).

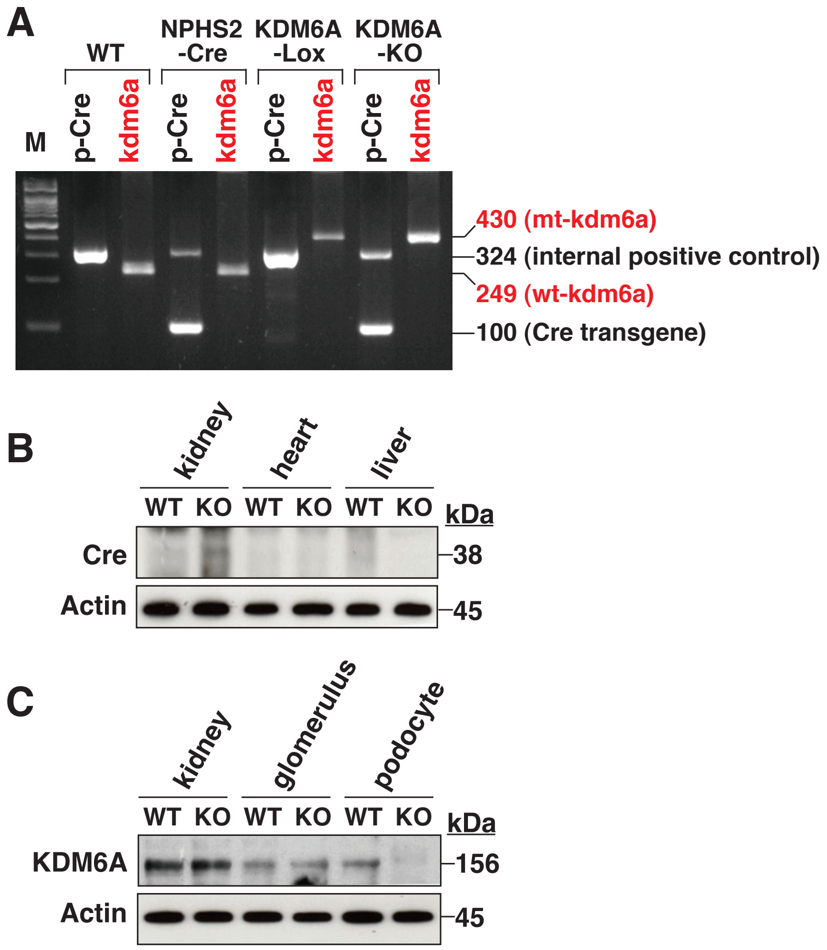

Appendix Figure S4 – Genotyping and expression of *KDM6A* in *KDM6A*-KO mice.

A Genotyping of podocyte-specific *KDM6A* knockout mice. Genomic DNAs were extracted from tail tissues of wild-type, transgenic Podocin-Cre [129S6.Cg-Tg(NPHS2-cre)259Lbh/BroJ], *KDM6*^{flox} (129S-Kdm6a$^{tm1.1Kaig}$) and *KDM6A*-KO mice. The presence of the *Cre* transgene was identified as a 100-bp fragment in PCR reaction, whereas the presence of the floxed *KDM6A* allele was identified as a 430-bp PCR fragment.

B Specific expression of Cre recombinase in kidney tissues of *KDM6A*-KO mice.

C Expression profiles of KDM6A in kidneys, glomeruli and podocytes isolated from wild-type and *KDM6A*-KO mice.

Table S1: Exact *P* values for Figure 1

Figure Panels	*P* value	Significance	Mark
**1A**_Nephrin levels			
HG vs. NC (24 hr)	*P*=0.0132	Yes	*
HG vs. NC (48 hr)	*P*=0.0061	Yes	*
HG vs. NC (72 hr)	*P*=0.0038	Yes	*
**1C**_Nephrin levels			
HG vs. NC	*P*=0.0182	Yes	*
HG (KDM-i) vs. HG	*P*=0.0061	Yes	#
**1E**_Levels of KDM mRNAs			
HG vs. NC (KDM3A)	*P*=0.1281	No	
HG vs. NC (KDM4A)	*P*=0.4631	No	
HG vs. NC (KDM5A)	*P*=0.3645	No	
HG vs. NC (KDM5B)	*P*=0.2364	No	
HG vs. NC (KDM6A)	*P*=0.0043	Yes	*
HG vs. NC (KDM6B)	*P*=0.46235	No	
**1F**_KDM6A levels			
HG vs. NC (24 hr)	*P*=0.0236	Yes	*
HG vs. NC (48 hr)	*P*=0.0092	Yes	*
HG vs. NC (72 hr)	*P*=0.0077	Yes	*
**1G**_Histone modifications			
HG vs. NC (H3K27me1)	*P*=0.7231	No	
HG vs. NC (H3K27me2)	*P*=0.0182	Yes	*
HG vs. NC (H3K27me3)	*P*=0.0082	Yes	*
HG vs. NC (meH3K9)	*P*=0.3817	No	

Table S2: Exact *P* values for Figure 2

Figure Panels	*P* value	Significance	Mark
**2A**_KDM6A knockdown			
HG (siCtrl) vs. NC			
KDM6A levels	*P*=0.0136	Yes	*
Nephrin levels	*P*=0.0127	Yes	*
WT-1 levels	*P*=0.0093	Yes	*
HG (siKDM6A) vs. NC			
KDM6A levels	*P*=0.0213	Yes	*
Nephrin levels	*P*=0.4873	No	
WT-1 levels	*P*=0.5162	No	
Mann (siKDM6A) vs. NC			
KDM6A levels	*P*=0.0134	Yes	*
Nephrin levels	*P*=0.6473	No	
WT-1 levels	*P*=0.7432	No	
HG (siKDM6A) vs. HG (siCtrl)			
KDM6A levels	*P*=0.0028	Yes	#
Nephrin levels	*P*=0.0076	Yes	#
WT-1 levels	*P*=0.0085	Yes	#
**2B**_KDM6A overexpression			
HG vs. NC			
KDM6A levels	*P*=0.0146	Yes	*
Nephrin levels	*P*=0.0086	Yes	*
WT-1 levels	*P*=0.0136	Yes	*
KDM6A vs. Vector			
KDM6A levels	*P*=0.0008	Yes	*
Nephrin levels	*P*=0.0027	Yes	*
WT-1 levels	*P*=0.0039	Yes	*

Table S2: Exact *P* values for Figure 2_(Continued)

**Figure Panels**	***P* value**	**Significance**	**Mark**
**2C**_H3K27me3 levels			
HG (siCtrl) vs. NC	*P*=0.0149	Yes	*
HG (siKDM) vs. HG (siCtrl)	*P*=0.0161	Yes	#
KDM6A vs. Vector	*P*=0.0073	Yes	*
**2E**_GSK-J4 treatment			
HG vs. NC			
Nephrin levels	*P*=0.0083	Yes	*
WT-1 levels	*P*=0.0115	Yes	*
H3K27me3 levels	*P*=0.0166	Yes	*
HG (GSK-J4) vs. HG (untreated)			
Nephrin levels	*P*=0.0058	Yes	#
WT-1 levels	*P*=0.0097	Yes	#
H3K27me3 levels	*P*=0.0146	Yes	#

Table S3: Exact *P* values for Figure 3

Figure Panels	*P* value	Significance	Mark
**3A**_STZ treatment (mRNA levels)			
DM vs. NC (KDM6A; 4 weeks)	*P*=0.0136	Yes	*
DM vs. NC (KDM6A; 8 weeks)	*P*=0.0032	Yes	*
DM vs. NC (KDM6A; 12 weeks)	*P*=0.0006	Yes	*
DM vs. NC (Nephrin; 4 weeks)	*P*=0.0122	Yes	*
DM vs. NC (Nephrin; 8 weeks)	*P*=0.0035	Yes	*
DM vs. NC (Nephrin; 12 weeks)	*P*=0.0043	Yes	*
DM vs. NC (WT-1; 4 weeks)	*P*=0.0055	Yes	*
DM vs. NC (WT-1; 8 weeks)	*P*=0.0071	Yes	*
DM vs. NC (WT-1; 12 weeks)	*P*=0.0016	Yes	*
**3B**_STZ treatment (protein levels)			
DM vs. NC (Nephrin; 4 weeks)	*P*=0.0132	Yes	*
DM vs. NC (Nephrin; 8 weeks)	*P*=0.0067	Yes	*
DM vs. NC (Nephrin; 12 weeks)	*P*=0.0036	Yes	*
DM vs. NC (KDM6A; 4 weeks)	*P*=0.0174	Yes	*
DM vs. NC (KDM6A; 8 weeks)	*P*=0.0064	Yes	*
DM vs. NC (KDM6A; 12 weeks)	*P*=0.0087	Yes	*
DM vs. NC (H3K27me3; 4 weeks)	*P*=0.0436	Yes	*
DM vs. NC (H3K27me3; 8 weeks	*P*=0.0126	Yes	*
DM vs. NC (H3K27me3; 12 weeks)	*P*=0.0116	Yes	*
**3D**_Biochemical tests			
Urinary protein levels			
DM vs. NC	*P*=0.0009	Yes	*
DM (GSK-J4) vs. DM	*P*=0.0045	Yes	#
Kidney weight (%)			
DM vs. NC	*P*=0.0127	Yes	*
DM (GSK-J4) vs. DM	*P*=0.0248	Yes	#
HbA1c (%)			
DM vs. NC	*P*=0.0082	Yes	*
DM (GSK-J4) vs. NC	*P*=0.0096	Yes	*
DM (GKS-J4) vs. DM	*P*=0.1044	No	

Table S3: Exact *P* values for Figure 3_(Continued)

Figure Panels	*P* value	Significance	Mark
**3E**_WB (GSK-J4 treatment)			
DM vs. NC			
KDM6A levels	*P*=0.0071	Yes	*
Nephrin levels	*P*=0.0101	Yes	*
WT-1 levels	*P*=0.0127	Yes	*
H3K27me3 levels	*P*=0.0143	Yes	*
DM (GSK-J4) vs. DM			
KDM6A levels	*P*=0.0098	Yes	#
Nephrin levels	*P*=0.0241	Yes	#
WT-1 levels	*P*=0.0149	Yes	#
H3K27me3 levels	*P*=0.0169	Yes	#
**3F**_IFA (Glomeruli)			
DM vs. NC			
KDM6A levels	*P*=0.0057	Yes	*
Nephrin levels	*P*=0.0036	Yes	*
WT-1 levels	*P*=0.0145	Yes	*
DM (GSK-J4) vs. DM			
KDM6A levels	*P*=0.0161	Yes	#
Nephrin levels	*P*=0.0058	Yes	#
WT-1 levels	*P*=0.0121	Yes	#

Table S4: Exact *P* values for Figure 4

Figure Panels	*P* value	Significance	Mark
**4A**_KDM6A mRNA levels			
KDM6A-KO vs. WT	*P*=0.0082	Yes	*
**4D**_Biochemical tests			
Urinary protein levels			
WT-DN vs. WT-NC	*P*=0.0092	Yes	*
KO-NC vs. WT-NC	*P*=0.7361	No	
KO-DM vs. WT-NC	*P*=0.0382	Yes	*
KO-DM vs. WT-DM	*P*=0.0082	Yes	#
Kidney weight (%)			
WT-DN vs. WT-NC	*P*=0.0142	Yes	*
KO-NC vs. WT-NC	*P*=0.5362	No	
KO-DM vs. WT-NC	*P*=0.4732	No	
KO-DM vs. WT-DM	*P*=0.0172	Yes	#
HbA1c (%)			
WT-DM vs. WT-NC	*P*=0.0048	Yes	*
KO-NC vs. WT-NC	*P*=0.6361	No	
KO-DM vs. WT-NC	*P*=0.0067	Yes	*
KO-DM vs. WT-DM	*P*=0.6143	No	
**4E**_IFA (Nephrin levels)			
WT-DM vs. WT-NC	*P*=0.0042	Yes	*
KO-DM vs. WT-DM	*P*=0.0141	Yes	#
**4F**_IFA (H3K27me3 levels)			
WT-DM vs. WT-NC	*P*=0.0136	Yes	*
KO-DM vs. WT-DM	*P*=0.0202	Yes	#
**4H**_WB (KDM6A-KO podocytes)			
Nephrin levels			
WT-DM vs. WT-NC	*P*=0.0073	Yes	*
KO-DM vs. WT-DM	*P*=0.0148	Yes	#
WT-1 levels			
WT-DM vs. WT-NC	*P*=0.0113	Yes	*
KO-DM vs. WT-DM	*P*=0.0209	Yes	#

Table S4: Exact *P* values for Figure 4_(Continued)

Figure Panels	*P* value	Significance	Mark
**4I**_WB (HG treatment)			
HG vs. NC			
Nephrin levels	*P*=0.0152	Yes	*
WT-1 levels	*P*=0.0201	Yes	*
Podocin levels	*P*=0.0172	Yes	*
Snail levels	*P*=0.7010	No	
KO (HG) vs. WT (HG)			
Nephrin levels	*P*=0.0225	Yes	#
WT-1 levels	*P*=0.0138	Yes	#
Podocin levels	*P*=0.0147	Yes	#
Snail levels	*P*=0.6486	No	

Table S5: Exact *P* values for Figure 5

Figure Panels	*P* value	Significance	Mark
**5B**_WB (lentivirus-KDM6A)			
KDM6A vs. Ctrl			
KDM6A levels	*P*=0.0076	Yes	*
Nephrin levels	*P*=0.0133	Yes	*
KLF10 levels	*P*=0.0156	Yes	*
Snail levels	*P*=0.7210	No	
**5C**_WB (HG treatment)			
HG vs. NC			
KDM6A levels	*P*=0.0133	Yes	*
KLF10 levels	*P*=0.0076	Yes	*
Nephrin levels	*P*=0.0058	Yes	*
**5D**_WB (KLF10 knockdown)			
KDM6A levels			
HG vs. NC	*P*=0.0216	Yes	*
HG (siCtrl) vs. NC	*P*=0.0178	Yes	*
HG (siKLF10) vs. HG (siCtrl)	*P*=0.0173	Yes	#
KLF10 levels			
HG vs. NC	*P*=0.0183	Yes	*
HG (siCtrl) vs. NC	*P*=0.0168	Yes	*
HG (siKLF10) vs. HG (siCtrl)	*P*=0.0173	Yes	#
Nephrin levels			
HG vs. NC	*P*=0.0079	Yes	*
HG (siCtrl) vs. NC	*P*=0.0276	Yes	*
HG (siKLF10) vs. HG (siCtrl)	*P*=0.0218	Yes	#
**5E**_IFA (GSK-J4 treatment)			
DM vs. NC			
KLF10 levels	*P*=0.0069	Yes	*
Nephrin levels	*P*=0.0133	Yes	*
DM (GSK-J4) vs. DM			
KLF10 levels	*P*=0.0101	Yes	*
Nephrin levels	*P*=0.0216	Yes	*

Table S5: Exact *P* values for Figure 5_(Continued)

Figure Panels	*P* value	Significance	Mark
**5F**_IFA (KDM6A-KO mice)			
KLF10 levels			
WT-DM vs. WT-NC	*P*=0.0116	Yes	*
KO-DM vs. WT-DM	*P*=0.0179	Yes	#
Nephrin levels			
WT-DM vs. WT-NC	*P*=0.0073	Yes	*
KO-DM vs. WT-DM	*P*=0.0102	Yes	*
**5G**_WB (GSK-J4 treatment)			
DM vs. NC			
KDM6A levels	*P*=0.0071	Yes	*
KLF10 levels	*P*=0.0080	Yes	*
Nephrin levels	*P*=0.0127	Yes	*
DM (GSK-J4) vs. DM			
KDM6A levels	*P*=0.0125	Yes	#
KLF10 levels	*P*=0.0083	Yes	#
Nephrin levels	*P*=0.0172	Yes	#
**5H**_WB (KDM6A-KO mice)			
KLF10 levels			
WT-DM vs. WT-NC	*P*=0.0128	Yes	*
KO-DM vs. WT-DM	*P*=0.0161	Yes	#
Nephrin levels			
WT-DM vs. WT-NC	*P*=0.0045	Yes	*
KO-DM vs. WT-DM	*P*=0.0116	Yes	#
**5I**_ChIP assay			
HG vs. NC (–2052/–1753 region)			
Anti-KLF10	*P*=0.0086	Yes	*
Anti-acetyl-H4	*P*=0.0145	Yes	*
Anti-Dnmt1	*P*=0.0043	Yes	*
Anti-Dnmt3	*P*=0.5281	No	

Table S5: Exact *P* values for Figure 5_(Continued)

Figure Panels	*P* value	Significance	Mark
**5I**_ChIP assay			
HG vs. NC (–1802/–1553 region)			
Anti-KLF10	*P*=0.0106	Yes	*
Anti-acetyl-H4	*P*=0.0258	Yes	*
Anti-Dnmt1	*P*=0.0047	Yes	*
Anti-Dnmt3	*P*=0.6781	No	
**5J**_WB (KLF10 overexpression)			
KLF10 vs. vector			
KLF10 levels	*P*=0.0094	Yes	*
KDM6A levels	*P*=0.0231	Yes	*
Nephrin levels	*P*=0.0118	Yes	*
WT-1 levels	*P*=0.0136	Yes	*
Podocin levels	*P*=0.0316	Yes	*
Synaptopodin	*P*=0.0146	Yes	*

Table S6: Exact *P* values for Figure 6

Figure Panels	*P* value	Significance	Mark
**6A**_KLF10 mRNA levels			
KLF10-KO vs. WT	*P*=0.00003	Yes	**
**6D**_Biochemical tests			
Urinary protein levels			
WT-DN vs. WT-NC	*P*=0.0085	Yes	*
KO-NC vs. WT-NC	*P*=0.8112	No	
KO-DM vs. WT-NC	*P*=0.0412	Yes	*
KO-DM vs. WT-DM	*P*=0.0154	Yes	#
Kidney weight (%)			
WT-DN vs. WT-NC	*P*=0.0216	Yes	*
KO-NC vs. WT-NC	*P*=0.7261	No	
KO-DM vs. WT-NC	*P*=0.4732	No	
KO-DM vs. WT-DM	*P*=0.0313	Yes	#
HbA1c (%)			
WT-DM vs. WT-NC	*P*=0.0079	Yes	*
KO-NC vs. WT-NC	*P*=0.5363	No	
KO-DM vs. WT-NC	*P*=0.0107	Yes	*
KO-DM vs. WT-DM	*P*=0.6477	No	
**6E**_IFA (Nephrin)			
WT-DM vs. WT-NC	*P*=0.0041	Yes	*
KO-DM vs. WT-DM	*P*=0.0073	Yes	#
**6F**_IFA (KDM6A)			
WT-DM vs. WT-NC	*P*=0.0086	Yes	*
KO-DM vs. WT-DM	*P*=0.0093	Yes	#
**6I**_WB (KLF10-KO mice)			
KDM6A levels			
WT-DM vs. WT-NC	*P*=0.0086	Yes	*
KO-DM vs. WT-DM	*P*=0.0114	Yes	#
Nephrin levels			
WT-DM vs. WT-NC	*P*=0.0113	Yes	*
KO-DM vs. WT-DM	*P*=0.0210	Yes	#

Table S6: Exact *P* values for Figure 6_(Continued)

Figure Panels	*P* value	Significance	Mark
**6I**_WB (KLF10-KO mice)			
WT-1 levels			
WT-DM vs. WT-NC	*P*=0.0229	Yes	*
KO-DM vs. WT-DM	*P*=0.0208	Yes	#
**6J**_WB (TGF-β1/siKLF10)			
TGF-β1 vs. NC			
KLF10 levels	*P*=0.0208	Yes	*
KDM6A levels	*P*=0.0139	Yes	*
Nephrin	*P*=0.0116	Yes	*
WT-1	*P*=0.0093	Yes	*
TGF-β1 (siCtrl) vs. NC			
KLF10 levels	*P*=0.0221	Yes	*
KDM6A levels	*P*=0.0322	Yes	*
Nephrin	*P*=0.0177	Yes	*
WT-1	*P*=0.0218	Yes	*
TGF-β1 (siKLF10) vs. TGF-β1 (siCtrl)			
KLF10 levels	*P*=0.0163	Yes	#
KDM6A levels	*P*=0.0225	Yes	#
Nephrin	*P*=0.0124	Yes	#
WT-1	*P*=0.0109	Yes	#

Table S7: Exact *P* values for Figure 7

Figure Panels	*P* value	Significance	Mark
**7A**_IFA (human)			
DN vs. Ctrl (KDM6A)	*P*=0.0131	Yes	*
DN vs. Ctrl (KLF10)	*P*=0.0136	Yes	*
DN vs. Ctrl (Nephrin)	*P*=0.0281	Yes	*
DM vs. Ctrl (WT-1)	*P*=0.0372	Yes	*
**7B**_WB (human)			
DN vs. Ctrl (KDM6A)	*P*=0.0139	Yes	*
DN vs. Ctrl (KLF10)	*P*=0.0152	Yes	*
DN vs. Ctrl (Nephrin)	*P*=0.0121	Yes	*
DM vs. Ctrl (WT-1)	*P*=0.0162	Yes	*
**7C**_Exosomal mRNA levels (human)			
DN vs. Ctrl (KDM6A)	*P*=0.0006	Yes	***
DN vs. Ctrl (KLF10)	*P*=0.0014	Yes	**
DN vs. Ctrl (Nephrin)	*P*=0.0255	Yes	*

Table S8: Exact *P* values for Figure EV1

Figure Panels	*P* value	Significance	Mark
**EV1A**_Urinary albumin levels			
WT-DM vs. WT-NC	*P*=0.0008	Yes	*
KO-DM vs. WT-NC	*P*=0.0128	Yes	*
KO-DM vs. WT-DM	*P*=0.0216	Yes	#
**EV1B**_Systolic blood pressure			
WT-DM vs. WT-NC	*P*=0.2618	No	
KO-DM vs. WT-NC	*P*=0.4328	No	
KO-DM vs. WT-DM	*P*=0.3821	No	
**EV1C**_Cystatin C levels			
WT-DM vs. WT-NC	*P*=0.4681	No	
KO-DM vs. WT-NC	*P*=0.4364	No	
KO-DM vs. WT-DM	*P*=0.6721	No	
**EV1D**_Tunnel assay			
WT-DM vs. WT-NC	*P*=0.0103	Yes	*
KO-DM vs. WT-NC	*P*=0.3125	No	
KO-DM vs. WT-DM	*P*=0.0216	Yes	#
**EV1E**_GBM thickness			
WT-DM vs. WT-NC	*P*=0.0098	Yes	*
KO-DM vs. WT-NC	*P*=0.1123	No	
KO-DM vs. WT-DM	*P*=0.0316	Yes	#
**EV1F**_PAS staining			
Glomeruli			
WT-DM vs. WT-NC	*P*=0.0102	Yes	*
KO-DM vs. WT-NC	*P*=0.1281	No	
KO-DM vs. WT-DM	*P*=0.0162	Yes	#
Tubules			
WT-DM vs. WT-NC	*P*=0.0068	Yes	*
KO-DM vs. WT-NC	*P*=0.0105	Yes	*
KO-DM vs. WT-DM	*P*=0.5211	No	

Table S9: Exact *P* values for Figure EV3

Figure Panels	*P* value	Significance	Mark
**EV3A**_Urinary albumin levels			
WT-DM vs. WT-NC	*P*=0.0029	Yes	*
KO-DM vs. WT-NC	*P*=0.0132	Yes	*
KO-DM vs. WT-DM	*P*=0.0331	Yes	#
**EV3B**_Systolic blood pressure			
WT-DM vs. WT-NC	*P*=0.3642	No	
KO-DM vs. WT-NC	*P*=0.6728	No	
KO-DM vs. WT-DM	*P*=0.5536	No	
**EV3C**_Cystatin C levels			
WT-DM vs. WT-NC	*P*=0.7368	No	
KO-DM vs. WT-NC	*P*=0.3447	No	
KO-DM vs. WT-DM	*P*=0.8231	No	
**EV3D**_Tunnel assay			
WT-DM vs. WT-NC	*P*=0.0088	Yes	*
KO-DM vs. WT-NC	*P*=0.4755	No	
KO-DM vs. WT-DM	*P*=0.0115	Yes	#
**EV3E**_GBM thickness			
WT-DM vs. WT-NC	*P*=0.0198	Yes	*
KO-DM vs. WT-NC	*P*=0.3123	No	
KO-DM vs. WT-DM	*P*=0.0233	Yes	#
**EV3F**_PAS staining			
Glomeruli			
WT-DM vs. WT-NC	*P*=0.0135	Yes	*
KO-DM vs. WT-NC	*P*=0.1891	No	
KO-DM vs. WT-DM	*P*=0.0144	Yes	#
Tubules			
WT-DM vs. WT-NC	*P*=0.0068	Yes	*
KO-DM vs. WT-NC	*P*=0.3310	No	
KO-DM vs. WT-DM	*P*=0.0116	Yes	#

Table S10: Exact *P* values for Figure EV4

Figure Panels	*P* value	Significance	Mark
**EV4A**_WB			
KDM6A vs. Vector			
KDM6A levels	*P*=0.0063	Yes	*
KLF10 levels	*P*=0.0016	Yes	*
Nephrin levels	*P*=0.0132	Yes	*
WT-1 levels	*P*=0.0094	Yes	*
KDM6A (siCtrl) vs. Vector			
KDM6A levels	*P*=0.0092	Yes	*
KLF10 levels	*P*=0.0102	Yes	*
Nephrin levels	*P*=0.0163	Yes	*
WT-1 levels	*P*=0.0048	Yes	*
KDM6A (siKLF10) vs. Vector			
KDM6A levels	*P*=0.0058	Yes	*
KLF10 levels	*P*=0.7202	No	
Nephrin levels	*P*=0.3673	No	
WT-1 levels	*P*=0.4807	No	
KDM6A (siKLF10) vs. KDM6A (siCtrl)			
KDM6A levels	*P*=0.7322	No	
KLF10 levels	*P*=0.0153	Yes	#
Nephrin levels	*P*=0.0214	Yes	#
WT-1 levels	*P*=0.0109	Yes	#
**EV4B**_WB			
KLF10 vs. Vector			
KDM6A levels	*P*=0.0188	Yes	*
KLF10 levels	*P*=0.0076	Yes	*
Nephrin levels	*P*=0.0107	Yes	*
WT-1 levels	*P*=0.0097	Yes	*

Table S10: Exact *P* values for Figure EV4_(Continued)

Figure Panels	*P* value	Significance	Mark
**EV4B**_WB			
KLF10 (siCtrl) vs. Vector			
KDM6A levels	*P*=0.0103	Yes	*
KLF10 levels	*P*=0.0082	Yes	*
Nephrin levels	*P*=0.0163	Yes	*
WT-1 levels	*P*=0.0148	Yes	*
KLF10 (siKDM6A) vs. Vector			
KDM6A levels	*P*=0.4603	No	
KLF10 levels	*P*=0.0124	Yes	*
Nephrin levels	*P*=0.0106	Yes	*
WT-1 levels	*P*=0.0098	Yes	*
KLF10 (siKDM6A) vs. KLF10 (siCtrl)			
KDM6A levels	*P*=0.0133	Yes	#
KLF10 levels	*P*=0.3536	No	
Nephrin levels	*P*=0.4351	No	
WT-1 levels	*P*=0.7739	No	

Table S11: Exact *P* values for Figure EV5

Figure Panels	*P* value	Significance	Mark
**EV5A**_WB			
HG vs. NC			
KDM6A levels	*P*=0.0067	Yes	*
KLF10 levels	*P*=0.0102	Yes	*
Nephrin levels	*P*=0.0086	Yes	*
WT-1 levels	*P*=0.0043	Yes	*
HG (anti-TGF-β1 Ab) vs. HG			
KDM6A levels	*P*=0.0096	Yes	#
KLF10 levels	*P*=0.0066	Yes	#
Nephrin levels	*P*=0.0112	Yes	#
WT-1 levels	*P*=0.0065	Yes	#
**EV5B**_WB			
KDM6A vs. Vector			
KDM6A levels	*P*=0.0028	Yes	*
KLF10 levels	*P*=0.0078	Yes	*
Nephrin levels	*P*=0.0062	Yes	*
WT-1 levels	*P*=0.0047	Yes	*
KDM6A (TGF-β1 Ab) vs. Vector (TGF-β1 Ab)			
KDM6A levels	*P*=0.0087	Yes	*
KLF10 levels	*P*=0.0069	Yes	*
Nephrin levels	*P*=0.0058	Yes	*
WT-1 levels	*P*=0.0033	Yes	*
KDM6A (TGF-β1 Ab) vs. KDM6A (untreated)			
KDM6A levels	*P*=0.5437	No	
KLF10 levels	*P*=0.6691	No	
Nephrin levels	*P*=0.7826	No	
WT-1 levels	*P*=0.4387	No	

Table S11: Exact *P* values for Figure EV5_(Continued)

Figure Panels	*P* value	Significance	Mark
**EV5C**_WB			
KLF10 vs. Vector			
KDM6A levels	*P*=0.0096	Yes	*
KLF10 levels	*P*=0.0027	Yes	*
Nephrin levels	*P*=0.0066	Yes	*
WT-1 levels	*P*=0.0082	Yes	*
KLF10 (TGF-β1 Ab) vs. Vector (TGF-β1 Ab)			
KDM6A levels	*P*=0.0099	Yes	*
KLF10 levels	*P*=0.0043	Yes	*
Nephrin levels	*P*=0.0106	Yes	*
WT-1 levels	*P*=0.0093	Yes	*
KLF10 (TGF-β1 Ab) vs. KLF10 (untreated)			
KDM6A levels	*P*=0.7685	No	
KLF10 levels	*P*=0.5521	No	
Nephrin levels	*P*=0.6587	No	
WT-1 levels	*P*=0.4372	No	

Table S12. Baseline characteristics of control subjects and patients with diabetic nephropathy

	Control (N = 12)	DM (N = 12)
Age (years)	46.4 ± 4.8	69.0 ± 8.2
Sex (male/female)	4/8	7/5
Diabetes types	−	type Ⅱ
eGFR (ml/min/1.73m^2)	90.1 ± 9.2	27.3 ± 17.9
TPCR (mg/g)*	65.5 ± 20.3	3260.1 ± 3370.1
HbA1c (%)	−	7.5 ± 1.7
Use of RAS blockers	0/12	10/12
Use of SGLT2 inhibitors	0/12	0/12
CVD#	0/12	4/12
CAD&	0/12	4/12

*TPCR: Total urine protein-to-creatinine ratio

#CVD: Cardiovascular disease

&CAD: Coronary artery disease
