# Supplementary material for: A KDM6A–KLF10 reinforcing feedback mechanism aggravates diabetic podocyte dysfunction
Source: EMBO Mol Med. 2019 Apr 4;11(5):e9828. doi: 10.15252/emmm.201809828 (PMC6505577; doi:10.15252/emmm.201809828)
Supplement: Supplementary file 2 — Expanded View Figures PDF [file EMMM-11-e9828-s002.pdf]

Expanded View Figures

Figure EV1. *In vivo* analysis of wild-type and podocyte-specific *KDM6A* knockout (*KDM6A*-KO) mice with or without STZ treatment.

A Urinary albumin excretion in wild-type and *KDM6A*-KO mice with or without STZ treatment. Urinary albumin levels were determined with a turbidimetric immunoassay (Autokit Micro Albumin, Wako, Osaka, Japan) at 8 weeks after diabetic induction. $^{*}P < 0.05$ versus untreated wild-type mice, $^{\#}P < 0.05$ versus STZ-treated wild-type mice (parametric ANOVA and a Bonferroni *post hoc* test; $n = 8$).

B Systolic blood pressure of wild-type and *KDM6A*-KO mice with or without STZ treatment. Systolic blood pressure was measured by tail–cuff plethysmography (BP-2000 Series II Blood Pressure Analysis System, Visitech Systems, Apex, NC, USA) at 8 weeks after diabetic induction. There were no statistical differences in mean systolic blood pressures between groups (parametric ANOVA and a Bonferroni *post hoc* test; $n = 8$).

C Levels of urinary cystatin C of wild-type and *KDM6A*-KO mice with or without STZ treatment. Urine cystatin C was measured using an ELISA kit (MSCTC0, R&D Systems) at 8 weeks after diabetic induction. No statistical differences in levels of urinary cystatin C were found between groups (parametric ANOVA and a Bonferroni *post hoc* test; $n = 8$).

D Detection of glomerular cell apoptosis in wild-type and *KDM6A*-KO mice with or without STZ treatment. TUNEL assay for apoptosis was performed using an assay kit according to the manufacturer's instruction (#TAAP01D, BioTnA Biotech., Kaohsiung Taiwan). Scale bars, 20 μm. $^{*}P < 0.05$ versus untreated wild-type controls, $^{\#}P < 0.05$ versus STZ-treated wild-type mice (parametric ANOVA and a Bonferroni *post hoc* test; $n = 3$).

E Representative electron micrographs of glomerular basement membrane (GBM) thickening and foot process effacement in wild-type and *KDM6A*-KO mice with or without STZ treatment. Kidney specimens for electron microscopy were prepared using a standard protocol described previously (White & Bilous, 2000; Advani *et al*, 2007), and electron micrographs were taken with a FEI Tecnai G2 F20 S-TWIN Transmission Electro Microscope (TEM). TEM images were processed and analyzed with DigitalMicrograph (Gatan Inc.). Scale bars, 0.5 μm. $^{*}P < 0.05$ versus untreated wild-type controls, $^{\#}P < 0.05$ versus STZ-treated wild-type mice (parametric ANOVA and a Bonferroni *post hoc* test; $n = 3$).

F Representative photographs of periodic acid–Schiff (PAS) staining of kidney tissues from wild-type and *KDM6A*-KO mice with or without STZ treatment. Scale bars, 20 μm. Levels of PAS staining in kidneys were quantified by integrated optical density (IOD) analysis. $^{*}P < 0.05$ versus untreated wild-type controls, $^{\#}P < 0.05$ versus STZ-treated wild-type mice (parametric ANOVA and a Bonferroni *post hoc* test; $n = 3$). As noted, diabetic *KDM6A*-KO mice revealed reduced PAS-staining intensity in renal glomeruli, but not in renal tubules, as compared to diabetic wild-type mice.

Data information: Data are expressed as mean ± SEM. See the exact *P*-values for comparison tests in Appendix Table S8.

© 2019 The Authors

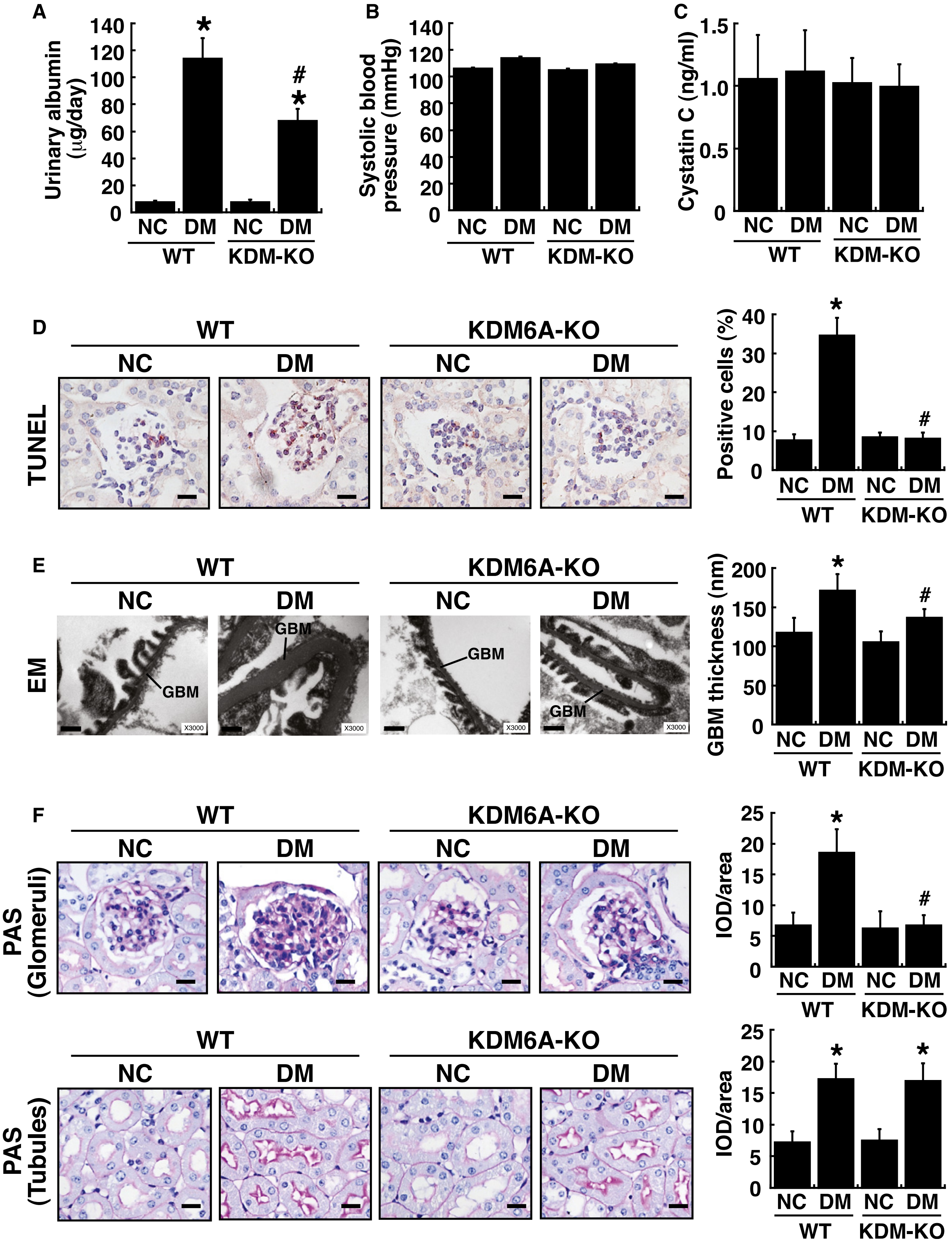
A
Urinary albumin (μg/day)
NC DM NC DM
WT KDM-KO
B
Systolic blood pressure (mmHg)
NC DM NC DM
WT KDM-KO
C
Cystatin C (ng/ml)
NC DM NC DM
WT KDM-KO
D
WT KDM6A-KO
NC DM NC DM
TUNEL
Positive cells (%)
E
WT KDM6A-KO
NC DM NC DM
EM
GBM
X3000
GBM thickness (nm)
F
WT KDM6A-KO
NC DM NC DM
PAS (Glomeruli)
IOD/area
PAS (Tubules)
IOD/area

Figure EV1.

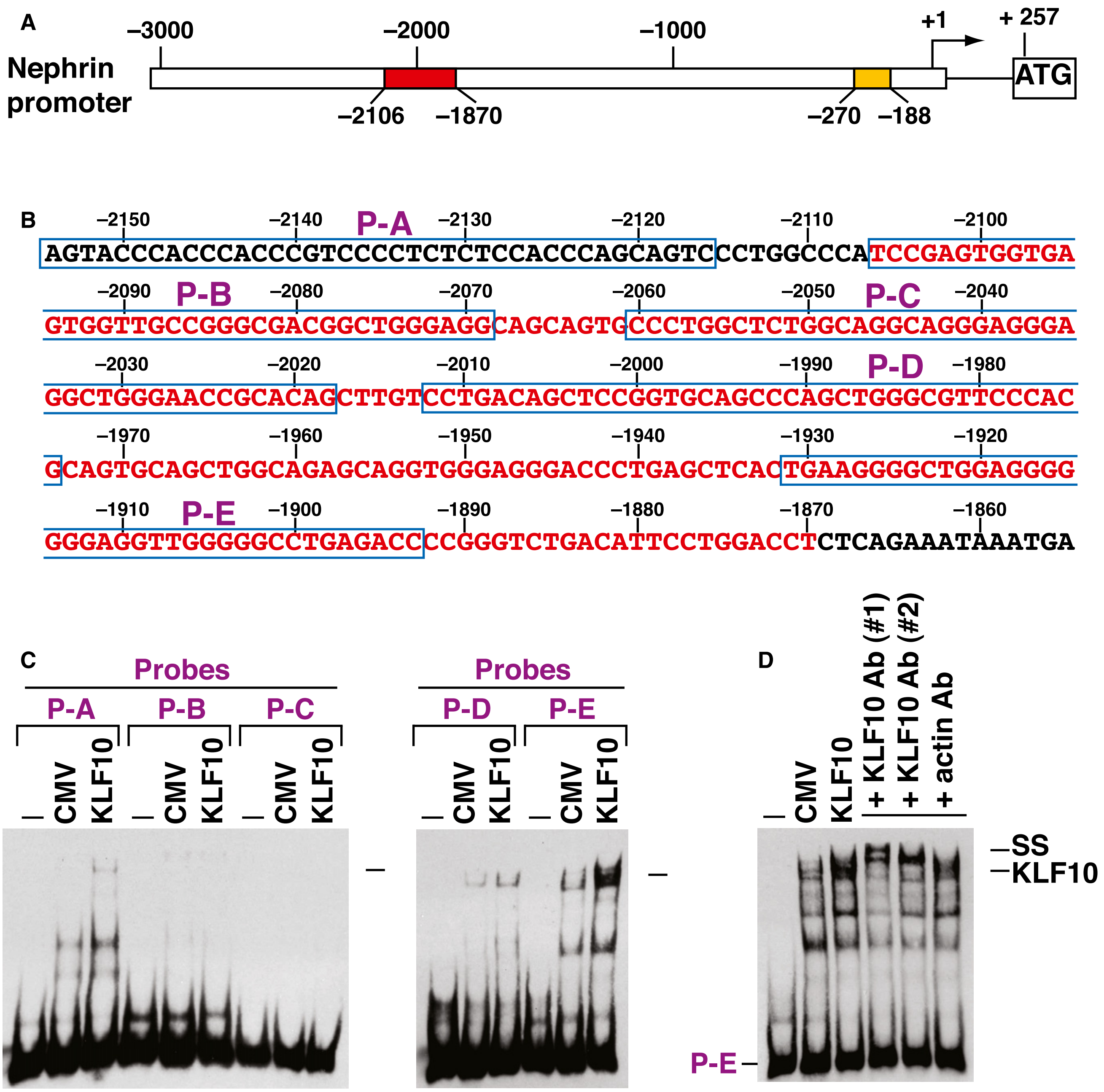

Figure EV2. EMSA analysis of KLF10 binding to the nephrin promoter region.

A Schematic diagram of mouse nephrin gene promoter. The transcriptional start site of mouse nephrin gene promoter (GenBank: AF190638.1) is located 257-bp upstream of the ATG codon. Two evolutionarily conserved promoter regions essential for podocyte-specific expression are indicated by yellow (from −188 to −270; 83-bp region) and red (from −1,870 to −2,106; 237-bp region) boxes, respectively. The 83-bp nephrin promoter element contains a WT-1 binding site, whereas the 237-bp promoter element contains multiple potential Sp1 binding sites.

B DNA sequence of the promoter region encompassing the 237-bp promoter region (from −1,870 to −2,106; red color) and the localization of five non-overlapping probes (P-A, P-B, P-C, P-D, and P-E) used in EMSAs.

C EMSAs of KLF10 binding to nephrin promoter elements. EMSA experiments were performed by using nephrin promoter elements (including P-A, P-B, P-C, P-D, and P-E) and protein lysates of 293T cells that were transfected with an empty vector or KLF10-expressing plasmid. Arrows indicated the potential KLF10/DNA complexes in EMSA experiments.

D Supershift analysis of the KLF10/DNA complex in EMSA. Different antibodies against KLF10 were used to supershift the formed complex in EMSA experiments using the P-E element as a probe. Anti-KLF10 antibody #1 was a kind gift from Dr. Vincent H.S. Chang, whereas anti-KLF10 antibody #2 (ab73537; Abcam) was obtained commercially.

 © 2019 The Authors

Figure EV3. *In vivo* analysis of wild-type and *KLF10*-knockout (*KLF10*-KO) mice with or without STZ treatment.

A Urinary albumin excretion in wild-type and *KLF10*-KO mice with or without STZ treatment. Urinary albumin levels were measured with a turbidimetric immunoassay (Autokit Micro Albumin, Wako, Osaka, Japan) at 8 weeks after diabetic induction. $^{*}P < 0.05$ versus untreated wild-type controls, $^{\#}P < 0.05$ versus STZ-treated wild-type mice (parametric ANOVA and a Bonferroni *post hoc* test; $n = 8$).

B Systolic blood pressure of wild-type and *KLF10*-KO mice with or without STZ treatment. Systolic blood pressure was measured by tail–cuff plethysmography (BP-2000 Series II Blood Pressure Analysis System, Visitech Systems, Apex, NC, USA) at 8 weeks after diabetic induction. No statistical differences in mean systolic blood pressures were found between groups (parametric ANOVA and a Bonferroni *post hoc* test; $n = 8$).

C Levels of urinary cystatin C of wild-type and *KLF10*-KO mice with or without STZ treatment. Urine cystatin C was measured using an ELISA kit (MSCTC0, R&D Systems) at 8 weeks after diabetic induction. No statistical differences in levels of urinary cystatin C were found between groups (parametric ANOVA and a Bonferroni *post hoc* test; $n = 8$).

D Detection of glomerular cell apoptosis in wild-type and *KLF10*-KO mice with or without STZ treatment. TUNEL assay for apoptosis was carried out using an assay kit according to the manufacturer's instruction (#TAAP01D, BioTnA Biotech., Kaohsiung Taiwan). Scale bars, 20 μm. $^{*}P < 0.05$ versus untreated wild-type controls, $^{\#}P < 0.05$ versus STZ-treated wild-type mice (parametric ANOVA and a Bonferroni *post hoc* test; $n = 3$).

E Representative electron micrographs of GBM (glomerular basement membrane) thickening and foot process effacement in wild-type and *KLF10*-KO mice with or without STZ treatment. Kidney specimens for electron microscopy were prepared as described previously (White & Bilous, 2000; Advani *et al*, 2007), and electron micrographs were taken with a FEI Tecnai G2 F20 S-TWIN Transmission Electro Microscope (TEM). TEM images were processed and analyzed with DigitalMicrograph (Gatan Inc.). Scale bars, 0.5 μm. $^{*}P < 0.05$ versus untreated wild-type controls, $^{\#}P < 0.05$ versus STZ-treated wild-type mice (parametric ANOVA and a Bonferroni *post hoc* test; $n = 3$).

F Representative photographs of periodic acid–Schiff (PAS) staining in kidney tissues of wild-type and *KLF10*-KO mice with or without STZ treatment. Scale bars, 20 μm. IOD: integrated optical density. $^{*}P < 0.05$ versus untreated wild-type controls, $^{\#}P < 0.05$ versus STZ-treated wild-type mice (parametric ANOVA and a Bonferroni *post hoc* test; $n = 3$). Notably, diabetic *KLF10*-KO mice substantially displayed reduced PAS-staining intensities in glomeruli and in renal tubules as compared to diabetic wild-type mice.

Data information: Data are expressed as mean ± SEM. See the exact *P*-values for comparison tests in Appendix Table S9.

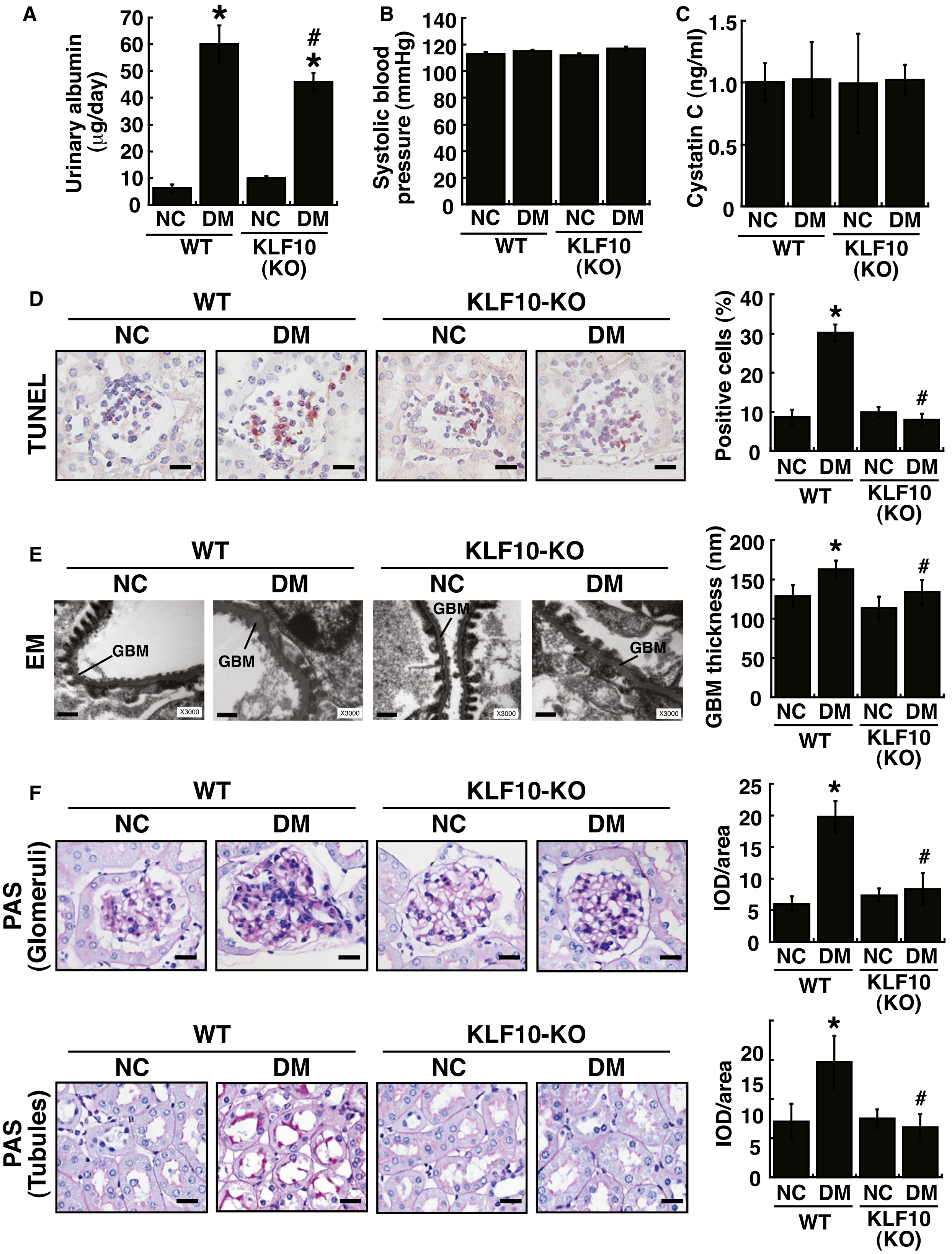

Figure EV3.

 © 2019 The Authors

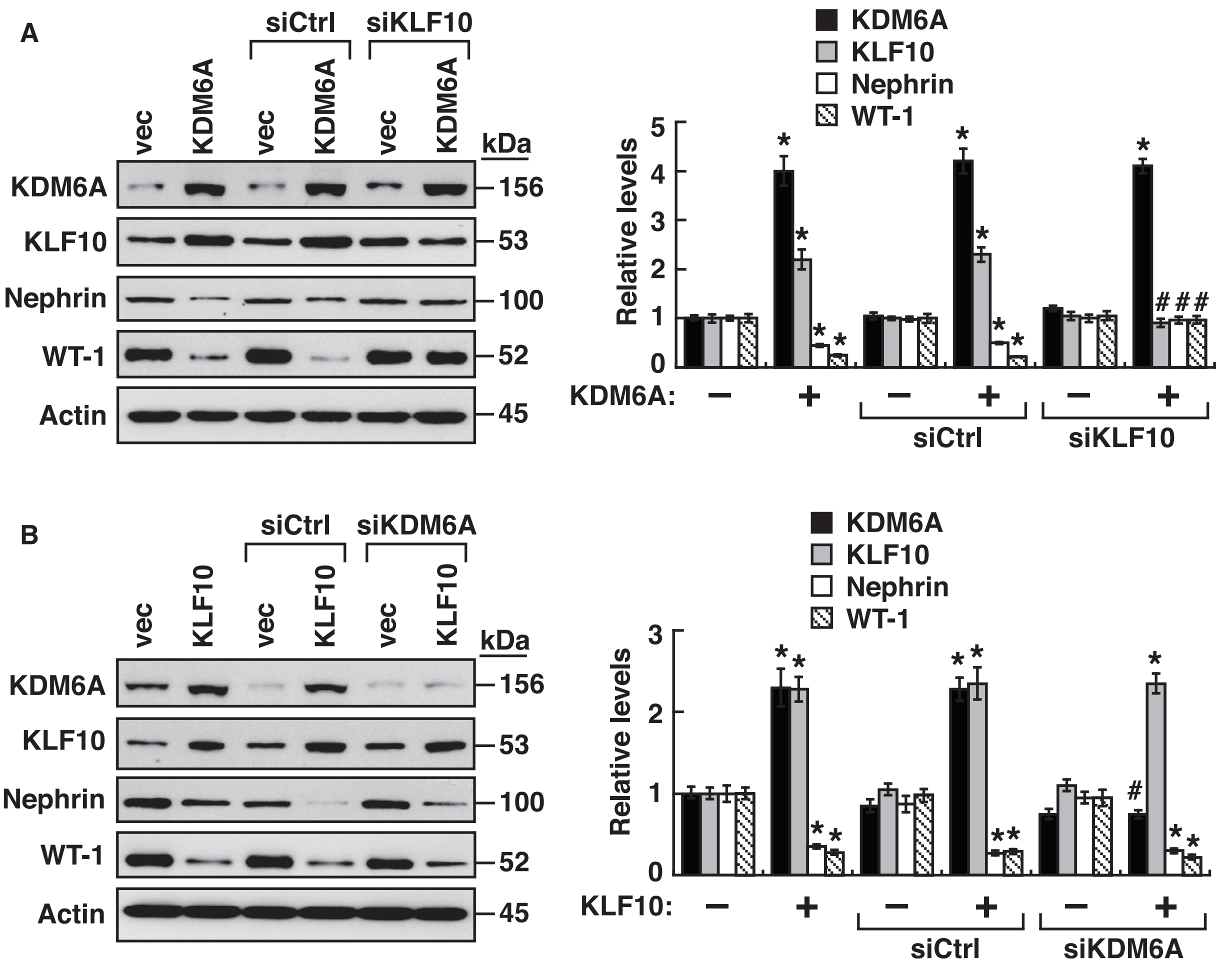

Figure EV4. KLF10 is dominant over KDM6A in mediating down-regulation of nephrin and WT-1.

A Effects of KDM6A overexpression in combination with KLF10 knockdown on the expression of podocyte-specific markers. Relative levels of KDM6A, KLF10, nephrin, and WT-1 expressed in podocytes that were transfected with the indicated plasmids, and siRNAs were determined by Western blot analysis. *$P < 0.05$ versus vector-transfected podocytes, $^{\#}P < 0.05$ versus podocytes transfected with KDM6A and control siRNA (parametric ANOVA and a Bonferroni *post hoc* test; $n = 3$).

B Effects of KLF10 overexpression in combination with KDM6A knockdown on the expression of podocyte-specific markers. Relative levels of KDM6A, KLF10, nephrin, and WT-1 expressed in podocytes that were transfected with the indicated plasmids and siRNAs were determined by Western blot analysis. *$P < 0.05$ versus vector-transfected podocytes, $^{\#}P < 0.05$ versus podocytes transfected with KLF10 and control siRNA (parametric ANOVA and a Bonferroni *post hoc* test; $n = 3$).

Data information: Data are expressed as mean ± SEM. See the exact *P*-values for comparison tests in Appendix Table S10.

Source data are available online for this figure.

© 2019 The Authors

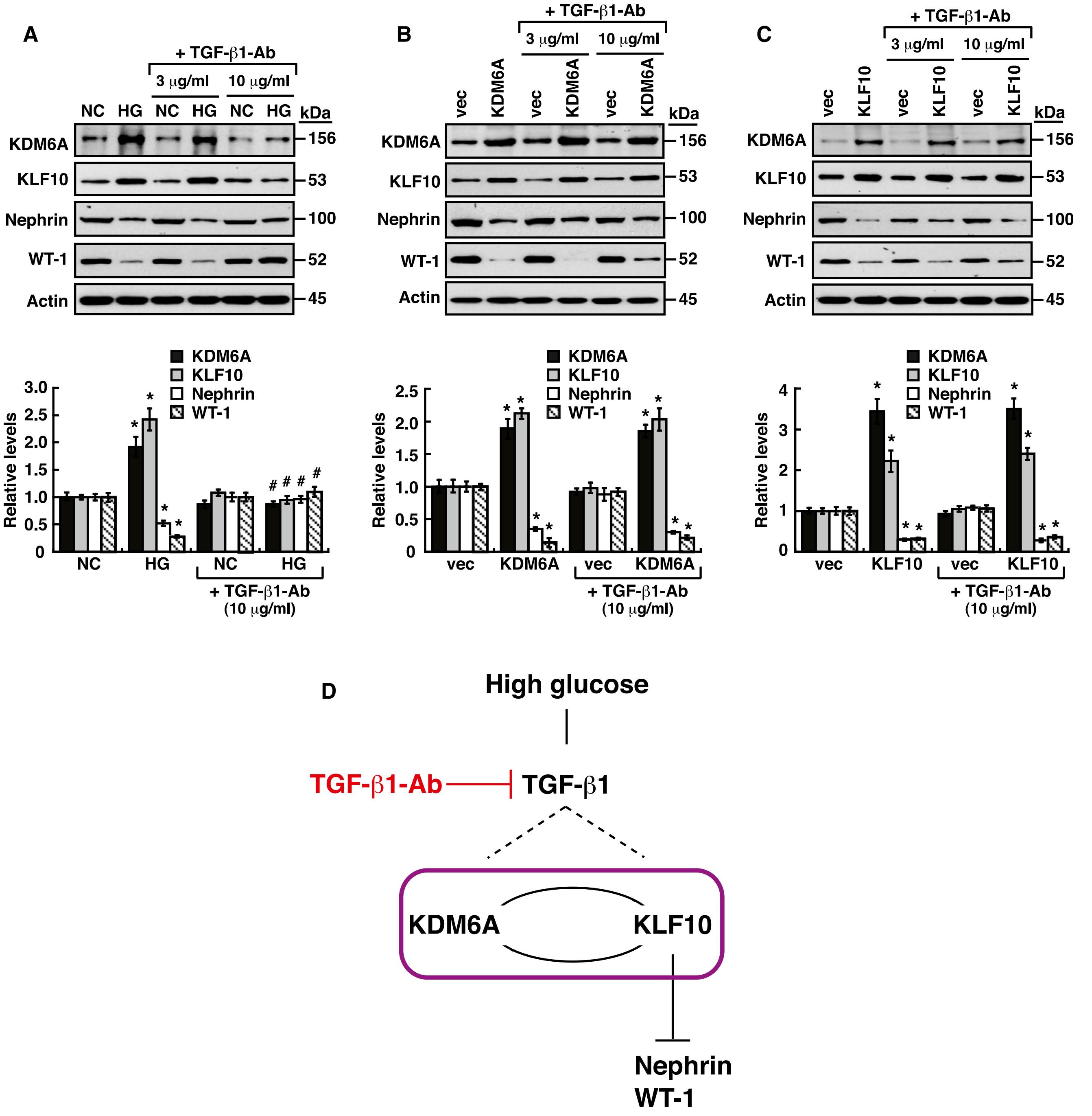

Figure EV5.

 © 2019 The Authors

Figure EV5. Addition of a TGF-β1-neutralizing antibody has no significant effects on the positive inter-regulation between KDM6A and KLF10 in podocytes.

A Effects of TGF-β1 neutralization on high glucose-mediated gene expression. Podocytes cultured in normal or high glucose were treated with different doses (3 and 10 μg/ml) of TGF-β1-neutralizing antibody (MAB240, R&D Systems) for 48 h. Relative protein levels of KDM6A, KLF10, nephrin, and WT-1 in these treated podocytes were analyzed by Western blot analysis. *$P < 0.05$ versus normal controls, $^{\#}P < 0.05$ versus podocytes in high glucose (parametric ANOVA and a Bonferroni *post hoc* test; $n = 3$).

B Effects of TGF-β1 neutralization on KDM6A-mediated gene expression. Podocytes transfected with an empty vector or KDM6A-expressing plasmid were treated with different doses (3 and 10 μg/ml) of TGF-β1 neutralizing antibody (MAB240, R&D Systems) for 48 h. Relative expression levels of KDM6A, KLF10, nephrin, and WT-1 in these treated podocytes were analyzed by Western blot analysis. *$P < 0.05$ versus podocytes transfected with an empty vector (parametric ANOVA and a Bonferroni *post hoc* test; $n = 3$).

C Effects of TGF-β1 neutralization on KLF10-mediated gene expression. Podocytes transfected with an empty vector or KLF10-expressing plasmid were treated with different concentrations (3 and 10 μg/ml) of TGF-β1 neutralizing antibody (MAB240, R&D Systems) for 48 h. Relative expression levels of KDM6A, KLF10, nephrin, and WT-1 in these treated podocytes were analyzed by Western blot analysis. *$P < 0.05$ versus podocytes transfected with an empty vector (parametric ANOVA and a Bonferroni *post hoc* test; $n = 3$).

D A model for the relationship between TGF-β1 signaling and the KDM6A–KLF10 positive feedback loop in podocytes under diabetic conditions. Under hyperglycemic conditions, increased TGF-β1 production may directly or indirectly trigger activation of the KDM6A–KLF10 positive feedback loop. However, TGF-β1 is not essential for the positive inter-regulation between KDM6A or KLF10.

Data information: Data are expressed as mean ± SEM. See the exact *P*-values for comparison tests in Appendix Table S11.

Source data are available online for this figure.

© 2019 The Authors
