# Supplementary figures and images for: A KDM6A–KLF10 reinforcing feedback mechanism aggravates diabetic podocyte dysfunction
Source: EMBO Mol Med. 2019 Apr 4;11(5):e9828. doi: 10.15252/emmm.201809828 (PMC6505577; doi:10.15252/emmm.201809828)

Fig EV4A

KDM6A

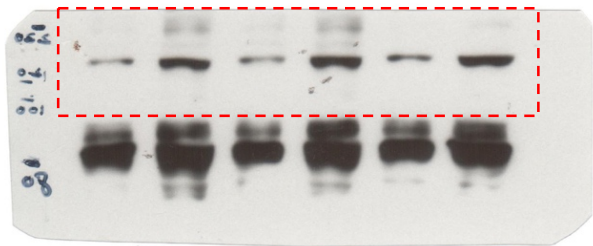

KLF10

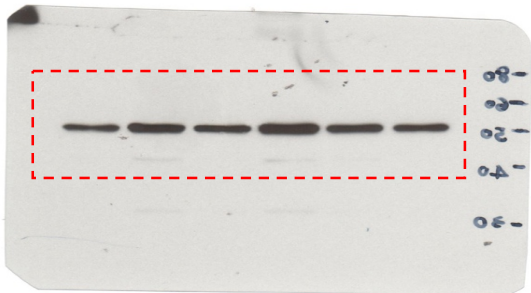

Nephrin

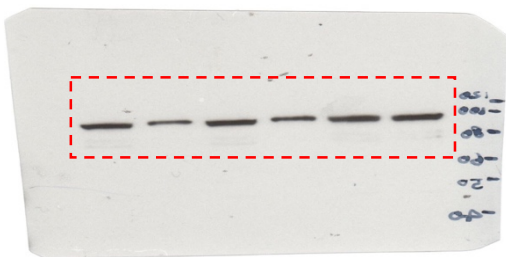

WT-1

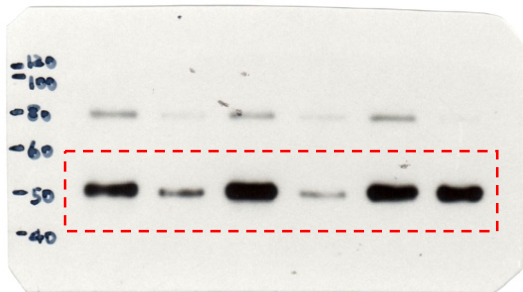

Actin

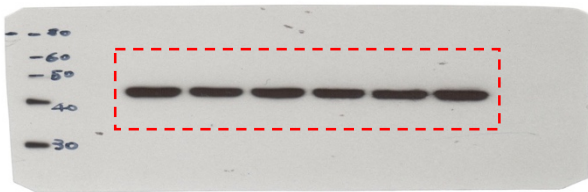

Fig EV4B

KDM6A

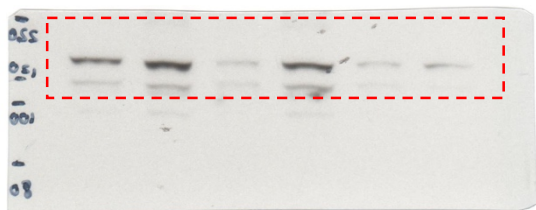

KLF10

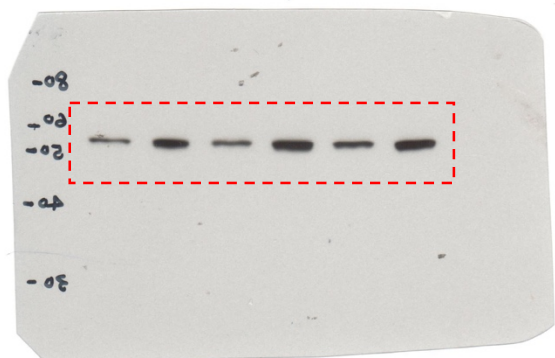

Nephrin

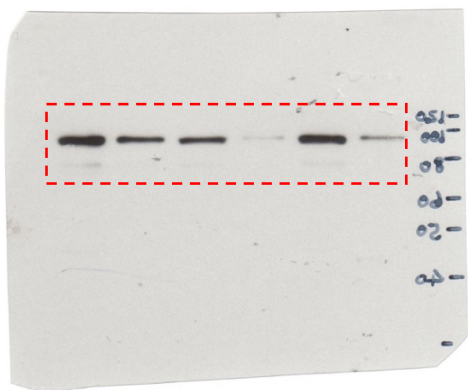

WT-1

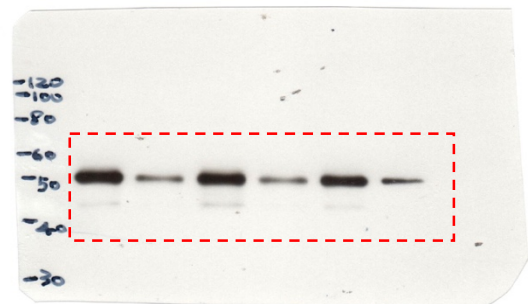

Actin

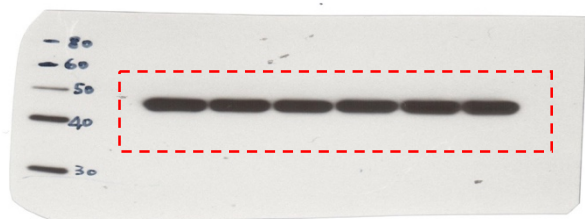

Supplement: Supplementary file 3 — Source Data for Expanded View [file EMMM-11-e9828-s003.zip › 9828_EV_source_data/Fig_EV4_(Source)-1.pdf]
